# Methanolic Extract of *Morinda citrifolia* L. (Noni) Unripe Fruit Attenuates Ethanol-Induced Conditioned Place Preferences in Mice

**DOI:** 10.3389/fphar.2016.00352

**Published:** 2016-09-27

**Authors:** Yasmin Khan, Vijayapandi Pandy

**Affiliations:** Department of Pharmacology, Faculty of Medicine, University of MalayaKuala Lumpur, Malaysia

**Keywords:** *Morinda citrifolia*, ethanol, conditioned place preference, dependence, extinction, reinstatement

## Abstract

Phytotherapy is an emerging field successfully utilized to treat various chronic diseases including alcohol dependence. In the present study, we examined the effect of the standardized methanolic extract of *Morinda citrifolia* Linn. unripe fruit (MMC), on compulsive ethanol-seeking behavior using the mouse conditioned place preference (CPP) test. CPP was established by injections of ethanol (2 g/kg, i.p.) in a 12-day conditioning schedule in mice. The effect of MMC and the reference drug, acamprosate (ACAM), on the reinforcing properties of ethanol in mice was studied by the oral administration of MMC (1, 3, and 5 g/kg) and ACAM (300 mg/kg) 60 min prior to the final CPP test postconditioning. Furthermore, CPPs weakened with repeated testing in the absence of ethanol over the next 12 days (extinction), during which the treatment groups received MMC (1, 3, and 5 g/kg, p.o.) or ACAM (300 mg/kg, p.o.). Finally, a priming injection of a low dose of ethanol (0.4 g/kg, i.p.) in the home cage (Reinstatement) was sufficient to reinstate CPPs, an effect that was challenged by the administration of MMC or ACAM. MMC (3 and 5 g/kg, p.o.) and ACAM (300 mg/kg, p.o.) significantly reversed the establishment of ethanol-induced CPPs and effectively facilitated the extinction of ethanol CPP. In light of these findings, it has been suggested that *M. citrifolia* unripe fruit could be utilized for novel drug development to combat alcohol dependence.

## Introduction

Alcoholism or alcohol dependence is defined as “a primary, continuing, recurrent brain disease with prolonged periods of frequent, heavy use, and a severe relapse even after long periods of abstinence” (Weiss, 2005). It occurs in a sequence that consists of: acute reinforcing effects of alcohol, transition from alcohol occasional use to alcohol compulsive use, and end-stage alcohol addiction (Cami and Farre, 2003). The common treatment strategies for chronic alcoholism adopted worldwide are comprised of psychotherapy (e.g., counseling) and pharmacotherapy (i.e., using detoxifying drugs such as disulfiram, naltrexone, and acamprosate; Miller and Gold, 1998). Yet, the chances of alcoholic reinstating alcohol consumption are extremely high, even after the individual has undergone available treatments (Aseltine et al., 2008). Within pharmacological approaches, some recent reports indicate the potential of complementary medicines (CMs) in the treatment of alcohol dependence (Mattioli et al., 2012). CMs are defined as the use of plant(s) and plant(s) based products for medicinal purposes as they can show efficacy with low toxicity (Lee et al., 2003; Sahraei et al., 2006). For example, various studies have highlighted the efficacies of medicinal herbs for the treatment of alcohol, nicotine, methamphetamine and opioids dependence (Lu et al., 2009) and some preclinical studies have indicated that *Hypericum perforatum* L., *Passiflora incarnata* L. *Pueraria lobata* Ohwi, *Salvia miltiorrhiza* Bge, *Tabernanthe iboga* H. Bn., and *Panax ginseng* hayer can be used to treat alcohol dependence (Abenavoli et al., 2009). Many psychoactive medicinal plants including *Morinda citrifolia* (mengkudu), *Salvia divinorum* (Diviner’s sage), *Mitragyna speciosa* (Kratom), and *Acorus calamus* (Jerangau/deringu) have been introduced into psychiatric practice in the past decades. Recently, *Morinda citrifolia* fruit has received wide attention globally and portrayed as a ‘miracle herb’ due to its variety of therapeutic benefits (Pandy et al., 2009, 2012).

*Morinda citrifolia* L. (Family: Rubiaceae) commonly known as “noni,” is a small tropical tree that grows widely in Polynesia. Noni fruit has been utilized as a food source in the tropical regions of the world (McClatchey, 2002). The different parts of this plant (e.g., fruit, leaf, bark, root, flower, and seed) have long been employed in folklore medicine to treat a broad range of diseases including diabetes, hypertension, arthritis, infections, gastric ulcers, depression, senility, menstrual difficulties, headaches, sprains, muscles aches, colds, cancer, poor digestion, atherosclerosis, blood vessels problems, and drug addiction (Samoylenko et al., 2006). Unfortunately, many of these claims regarding the efficacy of noni fruit to treat drug addictions have not been scientifically demonstrated in the literature. Recently, we reported the anticraving property of methanolic extract of *Morinda citrifolia* fruit against heroin dependence in rats (Narasingam et al., 2016) and we investigated the anticraving effect of Tahitian Noni^®^ Juice (TNJ) against ethanol seeking behavior in ICR mice using the CPP test (Pandy and Khan, 2016). In continuation to our recent reports, this study made a first attempt to establish the effectiveness of the noni fruit extract for treating alcohol dependence using a well-established animal model of drug reward, i.e., the CPP test.

The CPP paradigm is a widely used and validated animal model to study the rewarding effects of a wide variety of self-administered drugs, including ethanol (Tzschentke, 2007). It can also be used to model the relapse of drug-seeking behavior after a period of drug withdrawal or abstinence; in such instances, exposure to low doses of the addictive substance or the administration of mild stress can induce reinstatement of otherwise “extinguished” CPPs (Aguilar et al., 2009).

The aim of the present study was to evaluate the effects of the methanolic extract of *Morinda citrifolia* unripe fruit (MMC) on the rewarding properties of ethanol in the CPP test. The experiment described below examined the effects of MMC on the acquisition, extinction and reinstatement of ethanol-induced CPPs in mice.

## Materials and Methods

### Preparation of the Methanolic Extract of *M. citrifolia* Unripe Fruit

The standardized methanolic extract of *M. citrifolia* (MMC) unripe fruit was prepared using a cold extraction with sonication as described in our recent publications (Pandy et al., 2012, 2014). The details of the phytochemical profiling of the methanolic extract of *M. citrifolia* (MMC) unripe fruit has been reported in our earlier publication (Pandy et al., 2014). It has been reported that the major phytoconstituents of MMC were characterized and quantified as scopoletin (18.95 μg/mg) and rutin (1.66 μg/mg; Pandy et al., 2014). The dried solvent free standardized MMC was stored in a refrigerator at 4°C in an airtight container until further use.

### Animals

Male ICR mice (*n* = 8–9 per group; UKM, Kuala Lumpur, Malaysia) weighing 25–30 g were housed in polycarbonate cages (in groups of 4–5 animals) for at least 7 days before use and were maintained on a 12:00/12:00-h light/dark cycle (lights off at 7:00 pm), at room temperature (20–22°C), and a humidity of 45–60%. Standard food pellets and purified drinking water were supplied *ad libitum*. All animals were acclimatized and handled for at least 1 week before the start of the experiment. Utmost care was taken to minimize animal suffering and all experimental procedures were reviewed and approved by the Institutional Animal Care and Use Committee, Faculty of Medicine, University of Malaya, Kuala Lumpur (Approval No. 2013-12-03/PHAR/R/VP) for adherence to the guidelines of the National Research Council of the National Academies of the USA (Garber et al., 2011).

### Drugs

Ethanol (10% v/v) was obtained by dilution of 95% v/v ethanol (Copens Scientific, Malaysia) in sterile water for injection. Ethanol and normal saline were administered intraperitoneally. Acamprosate (Sigma-Aldrich, USA) and MMC were suspended in 1% w/v CMC solution and were administered orally. All drug solutions were prepared fresh prior to start of the experiment and administered in a constant volume of 10 ml/kg body weight of the animal.

### Conditioned Place Preference Apparatus

Place preference conditioning was performed as described previously by (Cunningham, 1995; Kuzmin et al., 2003) with minor modifications. The CPP test box was made of Plexiglass [45(L) cm × 15(W) cm × 15(H) cm] and was divided into two equal-sized compartments [20 cm × 15 cm × 15 cm] with small middle gray compartment [5 cm × 15 cm × 15 cm] by insertion of two removable Plexiglass partitions. The two compartments had distinct visual and tactile cues: one side of the compartment had black walls with white horizontal stripes attached white wire mesh on the floor (white compartment) while the other side had white walls with vertical black lines and smooth plexiglass black floor (black compartment). Matched removable Plexiglass partitions were used to close off each compartment. Transparent plexiglass lids allowed the observation of the animal’s behavior on a computer connected to a Logitech HD Webcam placed above the apparatus. Mice behavior was recorded on a computer and later scored by an experimenter who was blind as to each subject’s treatment status.

**FIGURE 1:**
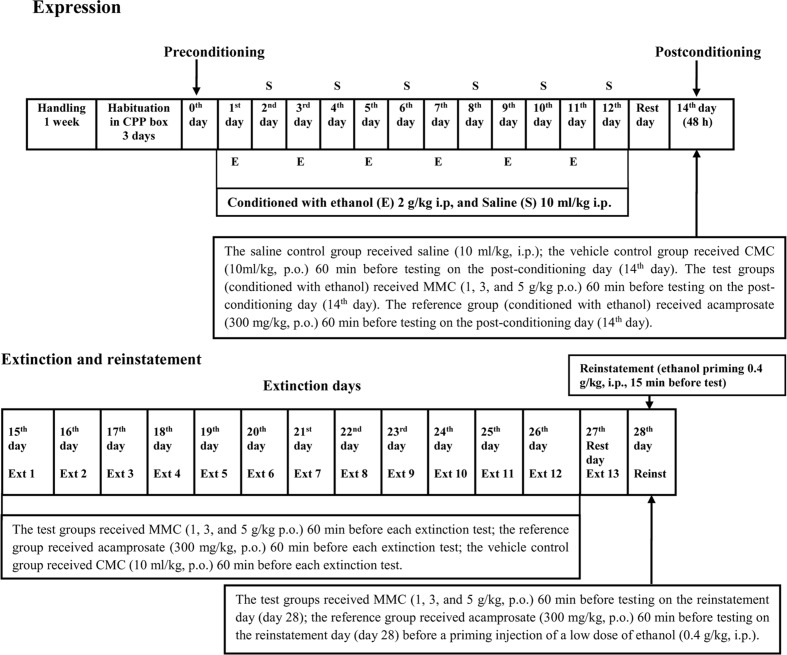
**Experimental timeline**.

The CPP methodology was divided into six distinct phases such as habituation, preconditioning/baseline, conditioning/ acquisition, postconditioning, extinction, and reinstatement, shown in **Figure 1**. All tests were conducted during the same time period of each day (0800–1500 h). The time schedule for the CPP test was modified to 12-day durations based on our pilot study in which we found that at least six injections of ethanol (2 g/kg, i.p.) were required to induce a significant CPP in ICR mice. Therefore, we followed this protocol and the same procedure was adopted in our recent published report in which we investigated the anticraving effect of Tahitian Noni^®^ Juice (TNJ) against ethanol seeking behavior in ICR mice using the CPP test (Pandy and Khan, 2016).

### Habituation and Preconditioning

All animals were habituated to the CPP apparatus for 3 days. On each habituation day each mouse was placed in the central gray compartment for 5 min and then both removable partitions were lifted thereby permitting the animals to explore freely throughout the apparatus for 15 min. The next day (Day 0) was conducted in the identical manner to habituation except that the time spent in each compartment was recorded and served as a preconditioning baseline. On every trial, animals were immediately returned to their home cages following testing. Any mouse that exhibited a strong unconditioned aversion (<33% of the session time spent in one side of the apparatus; i.e., 300 s) or preference (>66% of the session time spent in one side of the apparatus; i.e., 600 s) for any compartment was removed from the study. In the present study, white compartment was chosen as an ethanol-paired compartment as described elsewhere (Mattioli et al., 2012).

### Conditioning/Acquisition

Conditioning phase/acquisition was scheduled after the preconditioning session for next 12 days at 30-min daily conditioning sessions (Days 1–12). Saline and ethanol were injected on alternate days in alternate compartments (black and white) for 12 days. In the white compartment the mouse was injected with ethanol whereas in the black compartment, the mouse was injected with normal saline. Each mouse received ethanol injections (2 g/kg, i.p.) on days 1, 3, 5, 7, 9, and 11 and saline (10 ml/kg, i.p.) injections on days 2, 4, 6, 8, 10, and 12 of the conditioning phase. The saline control group received saline in both compartments on both odd and even days of 12-days conditioning/acquisition. On conditioning/acquisition days each mouse was placed in their respective compartments for 5 min and then were injected either saline or ethanol and confined to one side of the apparatus for 30 min after which the subjects were returned to their home cages.

### Postconditioning

The postconditioning (test day; Day 14) was scheduled 48 h later the last conditioning session. On day 14, the mice in ethanol free-state were placed in the center for 5 min and then both partitions were lifted and the animals allowed to move freely throughout the apparatus for 15 min as described for the baseline trial. The time spent in each compartment during the 15-min session was recorded. Conditioning scores in seconds were calculated by subtracting the time spent in the ethanol-paired white compartment to the time spent in saline-paired black compartment (the same was true for the saline-saline control group).

### Effect of MMC and ACAM on Expression of Ethanol-Induced CPP

To determine the effect of vehicle control (CMC), test extract (MMC), and reference drug, acamprosate (ACAM) on the expression of the rewarding properties of ethanol, different groups of mice (*n* = 9 for each group) was administered orally with 10 ml/kg of 1% w/v CMC or 1, 3, and 5 g/kg of MMC or 300 mg/kg of ACAM on the test day (Day 14), 60 min prior to the postconditioning test.

### Extinction of Ethanol CPP

Starting 24- h after the postconditioning test, the animals were given daily CPP testing that consisted of 15-min placements into the apparatus with free access to both compartments over each of the next 12 days (Days 15–26). Neither ethanol nor saline injections were given during this extinction period.

### Effect of MMC and ACAM on Extinction of Ethanol CPP

To determine the effects of CMC, MMC, and ACAM on extinction of CPP, the mice (*n* = 7–9 for each group) were first divided into two groups (saline and vehicle) to induce CPP (as described above). The CPP-induced vehicle group animals were further divided into CMC, MMC, and ACAM-treated groups. To determine whether CMC, MMC, and ACAM influenced the extinction time of ethanol-induced CPP, the mice were given extinction testing daily. Prior to each of the 12 CPP “extinction” trials, the mice (*n* = 7–9 for each group) in the vehicle control, test and reference groups were administered 10 ml/kg of 1% w/v CMC or MMC (1, 3, and 5 g/kg, p.o.) or ACAM (300 mg/kg p.o.) 60 min before testing. Behavioral testing was then conducted during 15-min sessions as described above (Ext 1–Ext 12).

### Effect of MMC and ACAM on a Low Dose Ethanol-Priming Reinstatement of CPP

To determine the effects of CMC, MMC, and ACAM on reinstatement of CPP, the mice were first divided into two groups to induce CPP (as described above), which received either saline or vehicle (10 ml/kg of 1%w/v CMC). They were then exposed to extinction testing to abolish ethanol-induced CPP. One day after the last extinction session, different groups of mice (*n* = 7–9 in each group) received 10 ml/kg of 1% w/v CMC or MMC (1, 3, and 5 g/kg, p.o.) or ACAM (300 mg/kg, p.o.) 60 min prior to a priming injection of a low dose of ethanol (0.4 g/kg, i.p.). These injections were administered in the colony room. After 15 min of priming injections, the mice were allowed free access to explore both compartments for 15 min, and the time spent in each compartment was measured.

### Statistical Analysis

All results are expressed as mean ± SEM. The CPP results were analyzed using two-way analysis of variance (ANOVA) followed by *post hoc* multiple comparisons using Bonferroni test and one-way ANOVA followed by a *post hoc* Newman-Keuls multiple comparison test. Statistical significance was set as *p* < 0.05.

## Results

### Effect of MMC and ACAM on Expression of Ethanol-Induced CPP

**FIGURE 2:**
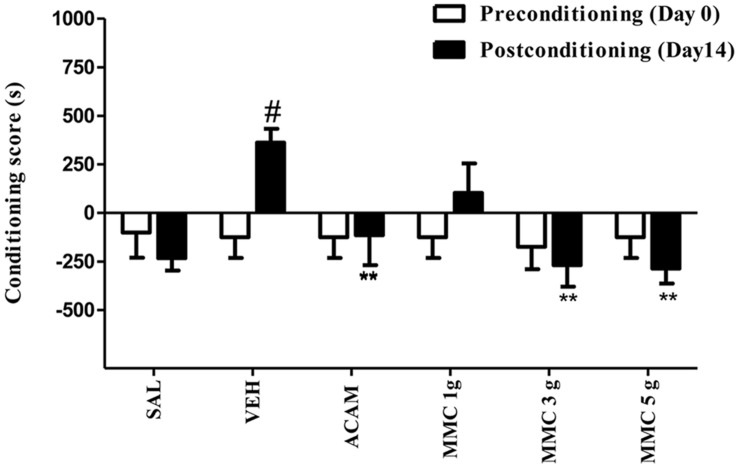
**Effect of MMC and ACAM on the expression of ethanol CPP in mice.** Data are expressed as the mean difference between the times spent in the compartment paired with ethanol and the times spent in the compartment paired with saline (*n* = 9/group). Significant difference #*P* < 0.05 was compared between preconditioning and postconditioning; ^∗∗^*p* < 0.01 was compared with the vehicle control group; when not indicated, the differences were not statistically significant.

The effect of MMC and ACAM on expression of ethanol-induced CPP is represented in **Figure 2**. ANOVA results revealed a significant effect of Group [*F*(5,96) = 2.76; *P* < 0.05] and the interaction (Group × Trial) [*F*(5,96) = 2.58; *P* < 0.05]. Bonferroni test revealed that the conditioning score of vehicle-treated group on postconditioning day was significantly (*p* < 0.05) increased when compared with the preconditioning day. However, the conditioning score on the postconditioning day was not altered in MMC (3 and 5 g/kg p.o.) and ACAM (300 mg/kg p.o.) treated groups. Interestingly, MMC (3 and 5 g/kg p.o.) and ACAM (300 mg/kg p.o.) significantly (*p* < 0.01) reduced the conditioning score when compared with the vehicle control group which implies the anticraving effect of MMC and ACAM.

### Effect of MMC and ACAM on Extinction of Ethanol CPP

**FIGURE 3:**
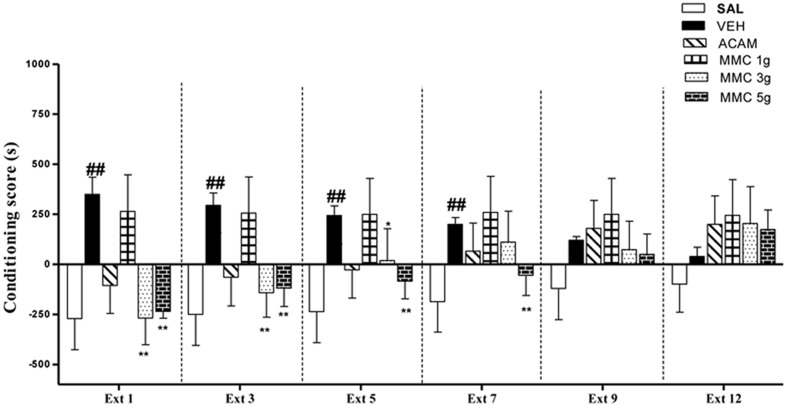
**Effect of MMC and ACAM on the extinction of ethanol CPP in mice.** Data are expressed as the mean difference between the times spent in the compartment paired with ethanol and the times spent in the compartment paired with saline (*n* = 7–9). Significant difference ##*p* < 0.01 was compared with the saline control group; ^∗^*p* < 0.05 and ^∗∗^*p* < 0.01 were compared with the vehicle control; when not indicated, the differences were not statistically significant.

The effect of MMC and ACAM on the extinction of ethanol-induced CPP is shown in **Figure 3**. ANOVA results revealed significant effects of Group [*F*(5,221) = 32.0; *P* < 0.001], Trial [*F*(5,221) = 4.16; *P* < 0.05], and a Group × Trial interaction [*F*(5,221) = 4.43; *P* < 0.001]. Separate One-way ANOVA on the data from each extinction trial revealed that there was a significant difference in conditioning score on extinction days: Ext 1, Ext 3, and Ext 5 [*F*(5,35) = 31.3, *p* < 0.001; *F*(5,35) = 9.28, *p* < 0.001; *F*(5,35) = 36.2, *p* < 0.001, respectively]. The vehicle control group showed a marked preference for the ethanol-paired compartment from Ext 1–7, after that it performed no differently than the saline control group. Interestingly, MMC (3 and 5 g/kg, p.o.), ACAM (300 mg/kg, p.o.) showed a marked accelerating effect on the extinction of ethanol CPP (**Figure 3**). The *post hoc* analysis revealed that MMC significantly reduced the conditioning score at a dose of 5 g/kg during extinction 1–7 and at a dose of 3 g/kg from extinction 1–5, when compared with the vehicle control group. Similarly, acamprosate (300 mg/kg, p.o.) treated animals showed significant reduction in conditioning score during extinction phase (Ext 1–5). However, MMC at a dose of 1 g/kg did not reduce the preference for the ethanol-paired environment across the entire extinction phase.

### Effect of MMC and ACAM on a Low Dose Ethanol Priming Reinstatement of CPP

**FIGURE 4:**
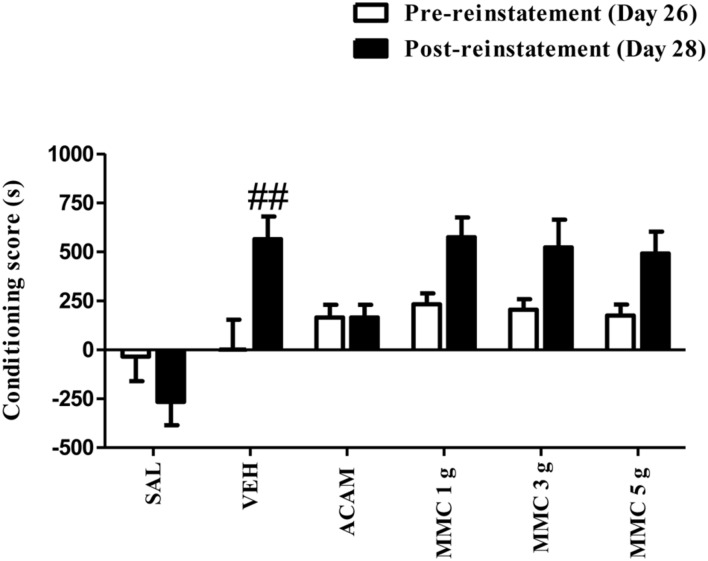
**Effect of MMC and ACAM on a low dose ethanol-priming reinstatement of CPP in mice.** Data are expressed as the mean difference between the times spent in the compartment paired with ethanol and the times spent in the compartment paired with saline (*n* = 7–9/group). Significant difference ##*p* < 0.01 was compared between pre-reinstatement and post-reinstatement; when not indicated, the differences were not statistically significant.

The effect of MMC and ACAM on a low dose ethanol priming-induced CPP is shown in **Figure 4**. ANOVA results revealed a significant effect of Group [*F*(5,75) = 7.86; *P* < 0.0001], Trial [*F*(1,75) = 12.7; *P* = 0.0006], and a Group × Trial interaction [*F*(5,75) = 3.76; *P* = 0.0043]. Bonferroni test revealed that the conditioning score of vehicle-treated group on the reinstatement day after a priming low dose of ethanol (0.4 g/kg, i.p.) was significantly (*p* < 0.05) increased when compared with the pre-reinstatement. Indeed, Bonferroni comparisons indicated no significant effect of MMC (1, 3, and 5 g/kg p.o.) or ACAM (300 mg/kg p.o.) on the reinstatement produced by the ethanol priming injection. Moreover, MMC and ACAM did not alleviate the conditioning score when compared with the vehicle control group.

## Discussion

The present study systematically investigated the effects of MMC and ACAM on the rewarding properties of ethanol as measured after different phases of place conditioning (i.e., acquisition/expression, extinction, and reinstatement). Each phase of the CPP test mimics a real clinical situation like expression for craving, extinction for abstinence, and reinstatement for relapse. The results of the present study provide evidence that: (1) intraperitoneal administration of ethanol (2 g/kg) can produce CPPs in mice as previously reported (Kurokawa et al., 2013); (2) the CPP can be weakened by repeated exposure to the place conditioning environment in an ethanol-free state; (3) a priming intraperitoneal administration of a low dose of ethanol (0.4 g/kg) successfully reinstated the CPPs as previously reported (Kuzmin et al., 2003); (4) MMC (3 and 5 g/kg, p.o.) and ACAM (300 mg/kg, p.o.) significantly weakened the expression of ethanol-induced CPP in mice, and (5) MMC and ACAM were found to facilitate the weakening of ethanol-induced CPPs during repeated extinction trials.

In general, the anticraving drugs available in the market to treat drug dependence (e.g., acamprosate) are able to attenuate acquisition and/or expression of drug-induced CPP (McGeehan and Olive, 2003; Lee et al., 2011; Kurokawa et al., 2013). In the present study, MMC and ACAM mitigated the expression of ethanol-induced CPPs indicating putative anticraving effects of MMC and ACAM. The major drawback of the CPP test is exhibiting false positive results for the drugs acting by simply impairing learning and memory without affecting drug reward pathways (e.g., NMDA receptor antagonists; Aguilar et al., 2009). Recently, Pachauri et al. (2012) demonstrated a nootropic effect of *Morinda citrifolia* fruit in which the fruit extract significantly reversed scopolamine-induced memory impairment in mice. Likewise, in another report, an ethyl acetate extract from noni fruit prevented beta-amyloid induced cognitive dysfunction in mice (Muralidharan et al., 2010). Therefore, the present attenuation of ethanol-induced CPPs by the MMC is unlikely to have been a result of a drug-induced impairment in memory processes.

Furthermore, Pachauri et al. (2013) demonstrated that there was no significant difference in spontaneous locomotor activity when mice were treated with a noni fruit extract. This report indicated that the current results cannot easily be accounted for by impairment in the subjects’ ability to move. Finally, we note that, an acute oral toxicity study of MMC was performed and described in our previous publication where no toxic effects of MMC were observed up to a dose of 20 g/kg (Pandy et al., 2012).

The results obtained from present study do not address the underlying neuronal or neurochemical mechanisms responsible for the efficacy of MMC in the expression and the extinction of ethanol-induced CPPs. Studies have shown that ethanol exerts its positive reinforcing effects primarily through the dopaminergic systems (reward pathways) that terminate in certain areas of the brain, such as the nucleus accumbens (Davis and Myers, 2002; Goodman, 2008; Aguilar et al., 2009). Administering any substances of abuse, eating (especially sweets), and sexual behavior or even gambling can increase intra synaptic levels of dopamine (DA) in the nucleus accumbens (Goodman, 2008). In our recent findings, antidopaminergic effect of MMC was established in *in vivo* and *ex vivo* studies (Pandy et al., 2012, 2014). Thus, we propose that the effects of MMC on the expression and facilitation of extinction of ethanol-induced CPPs may stem from its antidopaminergic activity.

Noni fruit has been claimed to have multifaceted therapeutic benefits due to its multiple phytoconstituents, such as scopoletin, rutin, quercetin, and kaempferol (Chan-Blanco et al., 2006). In our recent report, the major phytoconstituents of MMC were characterized and quantified as scopoletin (18.95 μg/mg) and rutin (1.66 μg/mg; Pandy et al., 2014). Furthermore, it has been suggested that scopoletin and rutin are responsible for the various pharmacological activities of noni including its antidopaminergic activity (Deng et al., 2007; Pandy et al., 2014). Although the present work could not delineate the active phytoconstituents that are responsible for the attenuation of ethanol-induced CPPs, we hypothesize that these behavioral effects are likely due to the anticraving effects of MMC. Clearly, further studies using scopoletin and rutin *per se* on ethanol-induced CPPs in experimental animals are warranted and are currently underway in our laboratory.

It has been previously demonstrated that re-exposure to a reinforcing drug or to stimuli associated with such a drug can reinstate the drug-seeking behaviors in animal models of drug abuse (Lu et al., 2003; Weiss, 2005). In the present study, a low dose of ethanol (0.4 g/kg bw, p.o.) priming in ethanol-extinguished animals significantly reinstated the ethanol seeking in the CPP test. However, all tested doses of MMC were found to be ineffective at reversing or attenuating the reinstatement of ethanol-induced CPPs following a low priming dose of ethanol.

## Conclusion

The present study demonstrates that MMC inhibited expression of and facilitated the extinction of ethanol-induced CPP in mice. These results suggest that MMC might act to attenuate the rewarding effects of ethanol and could therefore be utilized in the treatment of alcohol dependence. Nevertheless, though the discovery of an effective pharmacotherapy that addresses all aspects of alcohol dependence continues, and the present findings on MMC’s anticraving effect on ethanol-induced CPPs could motivate other researchers for further work.

## Author Contributions

YK performed the experiments, co-designed the study, accomplished the data analysis, and drafted the manuscript; VP conceived and designed the study and co-drafted the manuscript. Both authors have read and approved the final manuscript.

## Conflict of Interest Statement

The authors declare that the research was conducted in the absence of any commercial or financial relationships that could be construed as a potential conflict of interest.

## Abbreviations

ACAM: acamprosate
CMC: sodium carboxymethyl cellulose
CPP: Conditioned place preference
MMC: Methanolic extract of *Morinda citrifolia* unripe fruit
